# Depth or Breadth? Assessing the Impact of MusicWorks, a Creative Aging Music Program

**DOI:** 10.1093/geroni/igaf122.2136

**Published:** 2025-12-31

**Authors:** Katherine Richman, Julie Burkley, Caitlin Coyle

**Affiliations:** University of Massachusetts Boston, Boston, Massachusetts, United States; FriendshipWorks, Boston, Massachusetts, United States; University of Massachusetts Boston, Boston, Massachusetts, United States

## Abstract

Creative aging programs are essential for promoting cognitive health, social engagement, and emotional well-being among older adults. They provide opportunities for self-expression, help to combat isolation, and enhance memory. By fostering creativity through music, and storytelling, these programs empower older adults to stay active, connected and fulfilled. This presentation shares results from a mixed methods evaluation of MusicWorks, a program that uses music and music-related activities to create opportunities for residents of low-income buildings to socialize and interact with neighbors, thereby reducing isolation and loneliness. Through the integration of music-related activities (e.g. singing, dancing, music bingo), the program also seeks to promote cognitive stimulation and physical health. Data was collected via 9 focus groups (4 in Spanish) at 7 program sites around Boston (n = 92). Staff from 6 sites were also interviewed. Attendance records from 6 of the sites revealed that for most sites, there were first-time participants in attendance as well as those who attend regularly. There was some variation in terms of which sites had more frequent participation compared to others and this trend seemed to align with the frequency of programming (e.g., sites with monthly programming had less regular attendance compared to those with weekly programming). Results from the qualitative analysis suggest that music programming offers an outlet for connection as well as positive physical and cognitive outcomes. Deep social connections and endearment to the program comes with repeat participation, suggesting that depth is achieved by creating group dynamics. Facilitators and barriers to these outcomes will be discussed.

